# An investigation into the effects of band gap and doping concentration on Cu(In,Ga)Se_2_ solar cell efficiency

**DOI:** 10.1186/s40064-016-2256-8

**Published:** 2016-05-10

**Authors:** Md. Asaduzzaman, Mehedi Hasan, Ali Newaz Bahar

**Affiliations:** Department of Information and Communication Technology, Faculty of Engineering, Mawlana Bhashani Science and Technology University, Tangail, 1902 Bangladesh

**Keywords:** CIGS thin film, Absorber band gap, Doping concentration, Lattice mismatch, Efficiency

## Abstract

A simulation study of a Cu(In_1 − x_Ga_x_)Se_2_ (CIGS) thin film solar cell has been carried out with maximum efficiency of 24.27 % (V_oc_ = 0.856 V, J_sc_ = 33.09 mA/cm^2^ and FF = 85.73 %). This optimized efficiency is obtained by determining the optimum band gap of the absorber and varying the doping concentration of constituent layers. The Ga content denoted by x = Ga/(In + Ga) is selected as 0.35 which provides the optimum band gap of absorber layer as 1.21 eV. Theoretically, the effects of Ga fraction “x” on CIGS absorber band gap are investigated and to avoid the lattice mismatch effect, the efficiency measurements due to the CIGS band gaps >1.21 eV have not come to the consideration. A one-dimensional simulator ADEPT/F 2.1 has been used to analyze the fabricated device parameters and hence to calculate open circuit voltage, short circuit current, fill factor and efficiency.

## Background

The CIGS thin film hetero-junction solar cell based on the chalcopyrite p-type absorber layer Cu(In_1-x_Ga_x_)Se_2_ is a promising option in industrial productivity due to its lower manufacturing cost and higher efficiency (Rampino et al. 2015; Powalla and Dimmler 2001; Minemoto et al. 2003). Although the CIGS solar cell is recorded as a highly efficient (~21.7 %) thin film solar cell (Jackson et al. 2015) either there must still need to enhance efficiency and reduce cost for mass productivity. The inline co-evaporated CIGS absorber (Lindahl et al. 2013) has band gap range from 1.04 (CIS) to 1.67 eV (CGS) depending on x (from 0 to 1) (Tverjanovich et al. 2006; Gloeckler and Sites 2005; Gabor et al. 1996). The mismatch effect of CIGS layer (Lee et al. 2011) with adjacent CdS buffer layer and Mo back contact is avoided and the absorber band gap is adjusted with its corresponding electron affinity. Furthermore, the doping concentration of different layers is also an important factor to maximize the efficiency and minimize the fabrication cost of any solar cell (Haque and Galib 2013). The influence of the CIGS absorber band gap and the doping concentration of each layer on the performance of the solar cell have been investigated in this study. The radio frequency (RF) sputtered ZnO (deposition of Al doped ZnO and intrinsic ZnO) with its wider band gap of 3.3 eV and the chemical bath deposited (CBD) CdS with its direct band gap of 2.42 eV have been used as the window and the buffer layer respectively (Lindahl et al. 2013; Jung et al. 2010). All the efficiency measurements and comparisons are done under a solar spectrum AM1.5G for which the solar irradiance on earth is 0.1 W/cm^2^ (Haque et al. 2013). The shadowing factor used in the simulation is of 5 %.

## Research methodology

### Device modeling

**Fig. 1:**
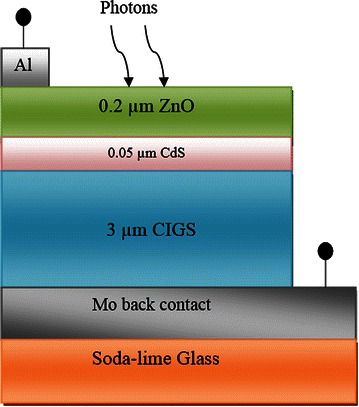
Schematic diagram of Cu (In_1-x_Ga_x_)Se_2_ thin film solar cell (The dimensions are not scaled)

ADEPT/F 2.1 (Gray et al. 2015), a one dimensional (1D) online research tool is used to analyze the device parameters as well as electrical characteristics of hetero-structured semiconductor devices including single, multi-junction and thin film solar cells. It contributes to the numerical solution of the Poisson’s equation and the continuity equation for holes and electrons. The simulator has been used to extract the diagrams such as electric field distribution, current-voltage characteristics, and energy band diagram. From the simulation, it becomes more feasible to calculate the fill factor (FF) and the efficiency. The CIGS model consisting of n-ZnO/n-CdS/p-CIGS layers fabricated on Mo coated soda-lime glass has been proposed for the simulation study. The structural view of Cu(In_1−x_Ga_x_)Se_2_ thin film solar cell used for conducting the simulation is shown schematically in Fig. 1.

### Methodological analysis

The simulation was conducted by formulating the parameters with their corresponding values used in Tables 1 and 2. Afterwards, the highest efficiency for this structure has been calculated by determining the optimum band gap of the absorber layer and making variation of the thickness and doping concentration of each layer. The simulations have been conducted through varying only the particular parameter and by keeping other parameters as default. The material and electrical properties of each layer have been elicited from some reliable sources of numerical simulations and experimental works (Gloeckler et al. 2003; Chelvanathan et al. 2010; Bouloufa et al. 2007; Schlenker et al. 2007; Balboul et al. 2008; Hossain et al. 2011; Repins et al. 2009; Gloeckler et al. 2005; Jackson et al. 2007). As mentioned earlier, the band gap of CIGS layer along with its electron affinity is varied according to the change in “x” content (Gorji et al. 2012). The absorption files used by the simulator define the absorption coefficient due to different wavelengths. These files and the values of the parameters corresponding to mid-gap defect states are extracted from (Gloeckler et al. 2003) for the constituent layers.

**Table 1:** The default values of device parameters

Parameters	N-ZnO	N-CdS	P-CIGS
Thickness (µm)	0.2	0.05	3
Dielectric constant	7.8	8.28	13.6
Refractive index	2	3.16	3.67
Band gap (eV)	3.3	2.42	1.15
Electron affinity (eV)	4.6	4.4	4.5
Electron mobility (cm^2^/Vs)	160	350	100
Hole mobility (cm^2^/Vs)	40	50	25
Conduction band effective density of states (cm^−3^)	2.2 × 10^18^	1.7 × 10^19^	2 × 10^18^
Valence band effective density of states (cm^−3^)	1.8 × 10^19^	2.4 × 10^18^	1.6 × 10^19^
Donor concentration (cm^−3^)	1 × 10^18^	1 × 10^18^	0
Acceptor concentration (cm^−3^)	0	0	2 × 10^16^
Electron lifetime (s)	5 × 10^−8^	2 × 10^−8^	1 × 10^−8^
Hole lifetime (s)	5 × 10^−9^	6 × 10^−8^	5 × 10^−8^
Absorption file	zno.a	cds.a	cigs.a

**Table 2:** Contact parameters for device simulation

Parameters	Front contact	Back contact
Reflectance	0.1	0.8
Recombination velocity for holes	10^7^	10^7^
Recombination velocity for electrons	10^7^	10^7^

**Fig. 2:**
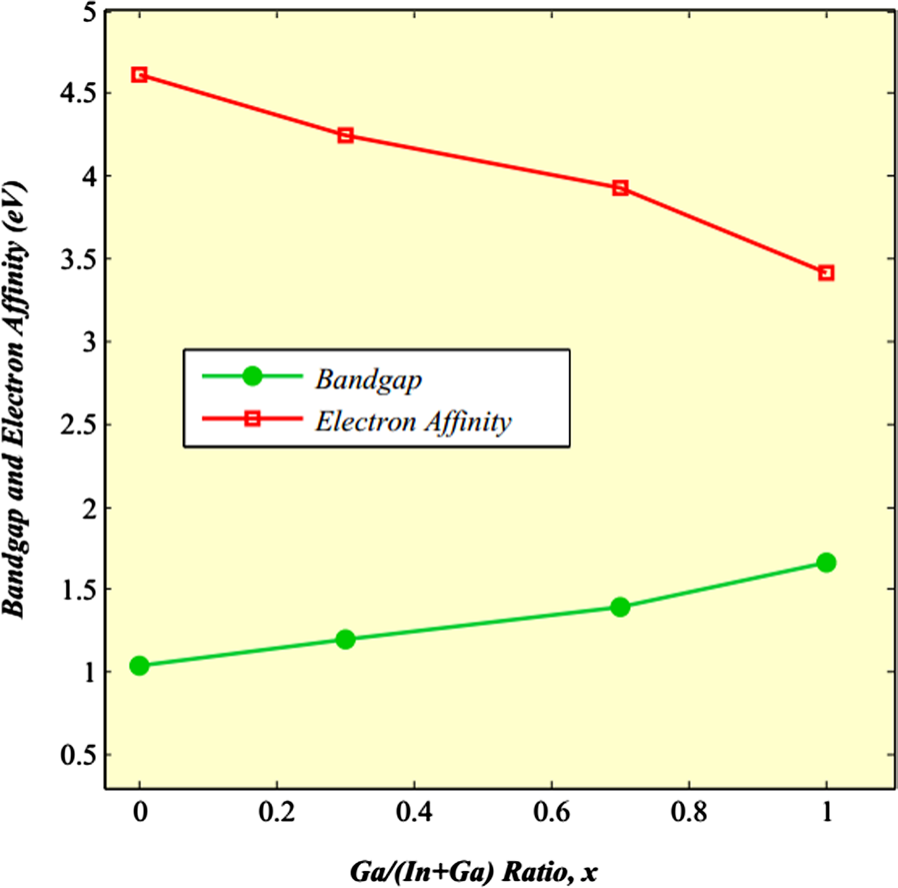
Variation in band gap and electron affinity due to the change in Ga content

The values of energy band gap and electron affinity are varied due to the change in Ga/(In+Ga) ratios. The Table 3 shows the variation in band gap along with electron affinity of Cu(In_1−x_Ga_x_)Se_2_ layer with respect to the variation in “x” (Song et al. 2004; Klein et al. 1996; Dejene 2009; Johnson 2004; Li et al. 2009; Minemoto et al. 2001; Gloeckler and Sites 2005; Černivec et al. 2007) which can successively be plotted through curve fitting as shown in Fig. 2.

**Table 3:** Band gap and electron affinity of Cu(In_1−x_Ga_x_)Se_2_ alloy composition

Ga/(In + Ga) ratio, x	Band gap, E_g_	Electron affinity, χ_e_
0.0	1.04	4.61
0.3	1.20	4.25
0.7	1.40	3.93
1.0	1.67	3.41

The mathematical Eqs. (1) and (2) were derived through fitting the curve by using the values put in Table 3.

1 $$E_{g} = 1.04 + 0.391x + 0.262x^{2}$$

2 $$\chi_{e} = 4.61 - 1.162x + 0.034x^{2}$$

It can be remarked that the band gap increases and the electron affinity decreases with the raising of “x”. Initially, the effect of absorber layer band gap was observed to determine the optimum result. Then, the energy band profile with optimum band gap and the efficiency graph due to the variation in energy gap were plotted. Afterwards, the doping concentration of each layer was varied and the optimal level of doping was determined by analyzing the corresponding efficiencies. Finally, the efficiency was calculated by using the optimized values and hence the highest performance was obtained.

## Results and discussion

### Simulation outcome with default values

**Fig. 3:**
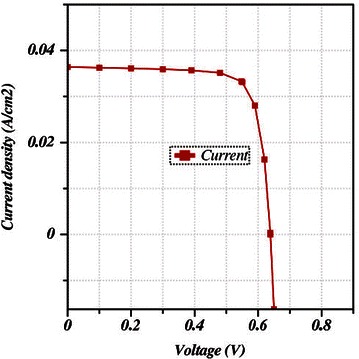
J-V characteristic curve for default values

The simulation was conducted successively with the default data used in Tables 1 and 2 which results in the light J-V characteristics curve. The open circuit voltage was obtained as 0.639 V, while the short circuit current was 36.41 mA/cm^2^. Then the fill factor (FF) and the efficiency were calculated as 78.38 and 18.23 % respectively. Figure 3 shows the J-V characteristic curve.

### Effect of absorber layer band gap on cell efficiency

The Cu(In_1−x_Ga_x_)Se_2_ layer energy band gap was varied from 1.04 to 1.69 eV and keeping the other parameters as default the corresponding efficiencies were calculated. However, the efficiency increases with the wider band gap and after certain level the efficiency decreases in spite of increasing the band gap. Furthermore, the lattice mismatch effect is an important issue to be noted in this case. The CIGS cell suffers from lattice mismatch effect for the Ga/(In + Ga) ratio above 0.35 (Song and Campbell 2013). The Table 4 shows the variation in band gap, electron affinity, and cell performance due to the change in Ga fraction. In a good agreement with simulation result, the optimal band gap of the CIGS absorber was chosen as 1.21 eV while the electron affinity was calculated as 4.21 eV. Because the band gap greater than 1.21 eV causes reducing the absorption within the layer and hence decreasing the short-circuit current. On the other side, the open circuit voltage increases linearly with the band gap variation.

**Table 4:** Performance variation due to absorber band gap

X	E_g_ (eV)	χ_e_ (eV)	J_sc_ (mA/cm^2^)	V_oc_ (V)	FF (%)	η (%)
0	1.04	4.61	36.43	0.529	76.70	14.79
0.2	1.13	4.38	36.22	0.622	77.68	17.49
0.35	1.21	4.21	36.06	0.705	76.44	19.45
0.55	1.33	3.98	35.84	0.828	66.60	19.77
0.7	1.44	3.81	35.69	0.940	55.90	18.43
1	1.69	3.48	35.45	1.183	32.16	13.49

**Fig. 4:**
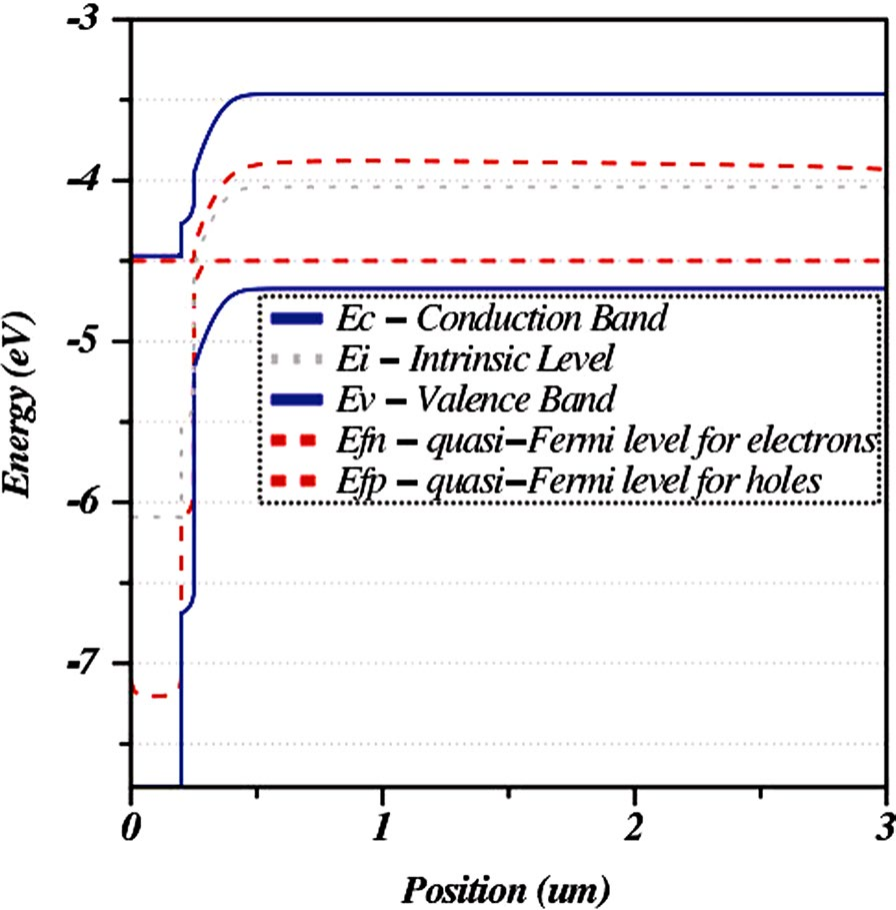
Energy band diagram of CIGS thin film

Determining the absorber band gap as 1.21 eV the energy band diagram for the entire solar cell structure was obtained as shown in Fig. 4.

### Effect of doping concentration on cell efficiency

The ZnO window layer doping concentration was varied from 1 × 10^17^ to 1 × 10^19^ cm^−3^ and the corresponding efficiencies were calculated to obtain the optimal doping level by comparing the outcomes. Hence, the optimal doping density was dictated as 1 × 10^18^ cm^−3^.

**Fig. 5:**
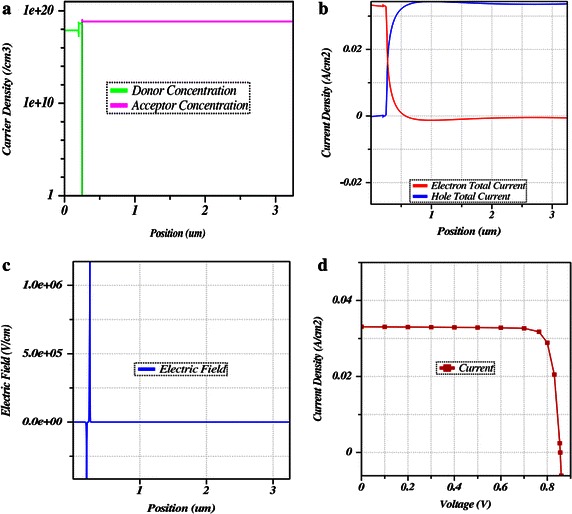
**a** Doping concentration **b** Spatially resolved current **c** Electric field and **d** J-V characteristic curve for optimum values

The doping density of CdS buffer on n-p junction (constituted between the buffer and the absorber) highly affects the output current. Analyzing the effects of drift velocity and holes recombination rate (Lee et al. 2009), the optimum doping concentration of CdS buffer layer was obtained as 5 × 10^18^ cm^−3^.

The determined higher doping level of the absorber, 1 × 10^19^ cm^−3^, is satisfactory for the electron affinity of the CIGS absorber, 4.21 eV.

### Optimized result

As discussed earlier, the absorber layer optimal band gap of 1.21 eV and the optimized doping concentration of all layers are determined through device simulation which in turn provides the highest performance. In Fig. 5a, describes the doping concentrations of different layers of designed CIGS solar cell, Fig. 5b shows the spatially resolved current, Fig. 5c denotes the electric field corresponding to the thickness of the layers and finally Fig. 5d represents the J-V characteristic curve from which the optimum efficiency has been calculated. The simulation result presents the J-V characteristic curve with short-circuit current density of 33.09 mA/cm^2^ and open circuit voltage of 0.856 V. Finally, the maximum efficiency of CIGS thin film was calculated from the simulation outcomes as 24.27 %.

## Conclusions

The numerical simulation of CIGS hetero-structure thin film solar cell was conducted by the ADEPT/F 2.1 one-dimensional online simulator. From various reliable sources the default values for simulation were collected and tabulated to obtain the default outcome. The mathematical equations of energy band gap and electron affinity for CIGS absorber as a function of “x” were developed by plotting some known results. At different Ga fraction the absorber band gap and electron affinity were calculated. The simulation of the cell with the Cu(In_0.65_Ga_0.35_)Se_2_ absorber layer results in higher efficiency rather than other compositions. Afterwards, the doping concentrations of the component layers were optimized in terms with drift velocity of the majority carrier and recombination rate of the minority carrier. At last, the cell performance was investigated by simulating with optimized values.
